# Effectiveness of written emotional disclosure interventions for eating disorders: a systematic review and meta-analysis

**DOI:** 10.3389/fnut.2024.1476956

**Published:** 2024-12-10

**Authors:** Yufei Wang, Tao Xu, Yuexian Tao, Xintong Cai

**Affiliations:** School of Nursing, Hangzhou Normal University, Hangzhou, China

**Keywords:** written emotional disclosure, expressive writing, eating disorders, body image, meta-analysis

## Abstract

**Background:**

Eating disorders are illnesses that can seriously impair the health and wellbeing of patients. Written emotional disclosure has been recognized as a therapeutic technique that may be helpful in aiding patients’ emotional and psychological adjustment. However, it is unclear what favorable effects eating disorder patients can derive from written emotional disclosure therapy. This study aimed to review the effectiveness of written emotional disclosure in treating eating disorders using a systematic review and meta-analysis.

**Objective:**

This study was to examine the validity of written emotional disclosure intervention for eating disorders as well as to provide guidelines for the management of eating disorders in patients.

**Methods:**

Researchers independently developed inclusion and exclusion criteria according to the PICOS principle and systematically searched English literature databases such as PubMed, Medline, Web of Science, Cochrane Library, CINAHL, EBSCO, Embase, and so on, from the time of library construction to December 2023. Cochrane Risk of Bias version 2 (RoB 2) was used to evaluate studies included in this review. All the studies included in this study were randomized controlled trials. Accurate information was extracted and then subjected to meta-analysis with Review Manager 5.4 software. The credibility of the evidence of the studies was assessed using GRADEprofiler 3.6 software.

**Results:**

The final analysis included 13 randomized controlled trials involving 1,444 participants. The written emotional disclosure intervention can decrease eating disorder symptoms scores (SMD = −0.20, 95% CI [−0.34, −0.05], *Z* = 2.59, *p* = 0.01), body dissatisfaction scores (SMD = 0.37, 95% CI [0.21, 0.52], *Z* = 4.59, *p* < 0.001), and thin ideal internalization score (SMD = 0.42, 95% CI [0.22, 0.62], *Z* = 4.12, *p* < 0.001). Anxiety scores (MD = 0.43, 95% CI [−0.77, 1.63], *Z* = 0.70, *p* = 0.48), depression scores (MD = −0.66, 95% CI [−1.78, 0.47], *Z* = 1.14, *p* = 0.25) and negative affect scores (SMD = 0.51, 95% CI [−0.24, 1.27], *Z* = 1.33, *p* = 0.18), with no statistically significant differences.

**Conclusion:**

In conclusion, this systematic review analyzed the existing literature and showed that written emotional disclosure intervention is effective in alleviating eating disorder symptoms and patients’ body image problems, but there is insufficient evidence in alleviating depression, anxiety, and negative affect. However, the evidence is limited. Therefore, more research is needed in the future to further enrich the evidence for written emotional disclosure intervention in the field of eating disorders.

**Systematic review registration:**

https://www.crd.york.ac.uk/prospero/,CRD42023445577.

## 1 Introduction

Eating disorders (EDs) are highly complex illnesses characterized by severe physical and psychological damage to the patient (1, 2). Due to the complexity of diagnosing eating disorders, their diagnostic definitions have been changing over the past decade (3, 4). The Diagnostic and Statistical Manual of Mental Disorders, Fourth Edition (DSM-IV) categorizes types of eating disorders as anorexia nervosa (AN), bulimia nervosa (BN), and eating disorders not otherwise specified (EDNOS) (5). Currently, the DSM-5 (6) incorporates binge-eating disorder (BED) into its overall classification of eating disorders, creating several new diagnosable eating disorders and expanding their definitions. Research indicates that unhealthy eating behaviors can lead to weight issues and disruptions in the endocrine system (7, 8). Additionally, individuals with eating disorders often experience feelings of loneliness or isolation, which can contribute to the development of more severe psychological problems (9). Furthermore, distorted body image perceptions can result in low self-esteem and diminished self-worth for those affected by eating disorders (10).

Also, eating disorders pose a significant threat to global health, and their prevalence is expected to increase. Statistics indicate that 3.3 million people worldwide lose their lives to ED every year (11). Studies have shown that ED can bring out a severe disability burden on society (12, 13). Moreover, according to Jared’s report (14), the estimated overall tangible economic burden of eating disorders in the United States amounts to $64.7 billion. The prevalence of eating disorders is high. A survey reported that the prevalence of eating disorders among adolescents in Ukraine, Hungary, and Poland was 37%, 22% and 20%, respectively (3). Some researchers in several reported Asian developing countries, such as Malaysia and Singapore, identified as high-risk populations at 50% and 43%, respectively, showed positive results on early screening for eating disorders (15, 16). And, the prevalence of ED is expected to increase. Several studies have shown that during the COVID-19 pandemic, eating disorders increased (17–20). In addition, based on a study by Taquet et al. (21), eating disorder diagnoses in the United States increased by 15.3% overall in 2020 compared with before COVID-19, and the relative risk has steadily increased since 2020. Therefore, disease management and prevention for people with eating disorders is particularly important.

The symptoms of eating disorders are serious and complex (22), and the types of medications applied in pharmacotherapy are also varied (23). Multifaceted early intervention may become indispensable to reduce the risk of long-term pathology and disability (24). Today, most interventions are focused on psychological, lifestyle, and physical activity (25). Regular high-intensity physical activity has been concluded to improve eating habits and overall health (26). A study by Irandoust et al. (27) proved that lifestyle interventions can be used as a long-term effective treatment modality for individuals with eating disorders. Additionally, Monaco et al. (28) have developed an advanced artificial intelligence platform to provide personalized treatment for patients with eating disorders. Although the current diversity of interventions all have favorable outcomes, psychotherapy is now recognized as a core intervention for the effective treatment of eating disorders (29). Currently, cognitive behavioral therapy (CBT-ED) (30) and low-intensity psychological interventions (31) have come a long way.

In recent years, expressive writing interventions have received increasing attention in the therapy of eating disorders. Expressive writing (EW), also known as written emotional disclosure (WED), is a succinct psychosocial intervention with the potential to improve psychological regulation (32). The characteristic of WED lies in its private nature, allowing individuals to explore their inner emotions without external interference. It is a structured approach to writing that facilitates cognitive and emotional processing of negative experiences and personal challenges, and aims to enhance the process of making meaning by creating narratives about their experiences, thereby prompting individuals to process their emotions and thoughts about traumatic experiences (33).

Regarding the potential effects of WED, it was not until the mid-1990s that a consistent literature began to emerge in the health, clinical, and psychosocial fields confirming the effectiveness of expressive writing in improving health (34). Researchers and medical professionals believe that using this strategy can help people improve their mental health, reduce eating disorder symptoms, and gain emotional control (35). In addition, there is evidence (36) that it can alleviate negative body image issues and develop self-compassion. Currently, in addition to traditional face-to-face writing therapy, remote web-based writing interventions have been developed (37). This improvement has expanded the target population for the intervention by increasing the convenience and effectiveness of the treatment.

Researches have shown that WED can reduce specific symptoms associated with eating disorders such as restricted eating and body dissatisfaction (38–40). However, some studies have shown that there was no significant effect of WED on body mass index or image concerns (41, 42). Lafont et al. (81) showed that WED only improved body image in patients with severe symptoms of eating disorders. Also, a study by Gamber et al. (43) concluded that WED did not reduce eating disorder behaviors or cognitions. It is unclear what favorable effects eating disorder patients can derive from written emotional disclosure therapy. Additionally, there is a lack of sufficient meta-analyses to evaluate the effectiveness of this treatment. Consequently, this study aimed to evaluate the efficacy of written emotional disclosure therapy for eating disorders by conducting a systematic review and meta-analysis.

## 2 Methods

This study was a systematic review and meta-analysis. The study follows the the guidelines for reporting systematic reviews and meta-analysis (44), specific PRISMA-2020 checklist is in Supplementary Table 3.

### 2.1 Inclusion and exclusion criteria

Criteria for inclusion included: (1) Individuals diagnosed with an eating disorder, such as anorexia nervosa, bulimia nervosa, or binge eating disorder according to the DSM-IV or DSM-5 classification systems, or individuals with high-risk factors for the development of eating disorders (e.g., internalization of body ideals, body image concerns, stress, cognitive flexibility deficits and so on) and participating in a written emotional disclosure intervention; (2) Participants of any age, gender or ethnicity; (3) Participants involved in the intervention engaged in expressive writing that focused on emotions and thoughts associated with eating disorder experiences; (4) Studies comparing written emotional disclosure interventions with no intervention/waiting list controls, conventional treatment controls, alternative psychological interventions (e.g., CBT, IPT), placebo, or attentional control interventions; (5) Randomized controlled trials (RCTs) comparing written emotional disclosure intervention to a control group. Clinical trials that are not randomized, including quasi-experimental and controlled trials.

Criteria for exclusion included: (1) Participants with comorbid psychiatric conditions unless data is reported separately for those with eating disorders only; (2) Studies evaluating interventions other than written emotional disclosure (e.g., CBT, IPT, medication); (3) Reviews, case studies, opinion pieces and non-empirical studies. (4) The study population included individuals with unspecified psychiatric or eating disorders, individuals with unspecified risk factors for eating disorders (people with ED-NOS will be excluded), or participants involved in treatment other than written emotional disclosure; (5) Studies that have deviation of intervention mode (e.g., studies that de-emphasized writing about affective experiences of eating disorders, focused on verbal disclosure rather than written disclosure, or did not use writing as a primary intervention component).

### 2.2 Search strategy

A systematic search was conducted across the following databases: PubMed, Medline, Cochrane Library, Web of Science, CINAHL, EBSCO, and Embase. The search included records from the inception of these major databases up to December 31, 2023.

We entered the following search terms in the above database: writing or “expressive writing” or “written disclosure” or “emotional expression” or “emotional disclosure” or “written emotional disclosure” or dairy or self-compassion* and “eating disorder” or “binge eating” or eating disorder* or eating disorders *. Supplementary Table 1 contains specific search formulas for each database.

### 2.3 Study selection

**Figure 1:**
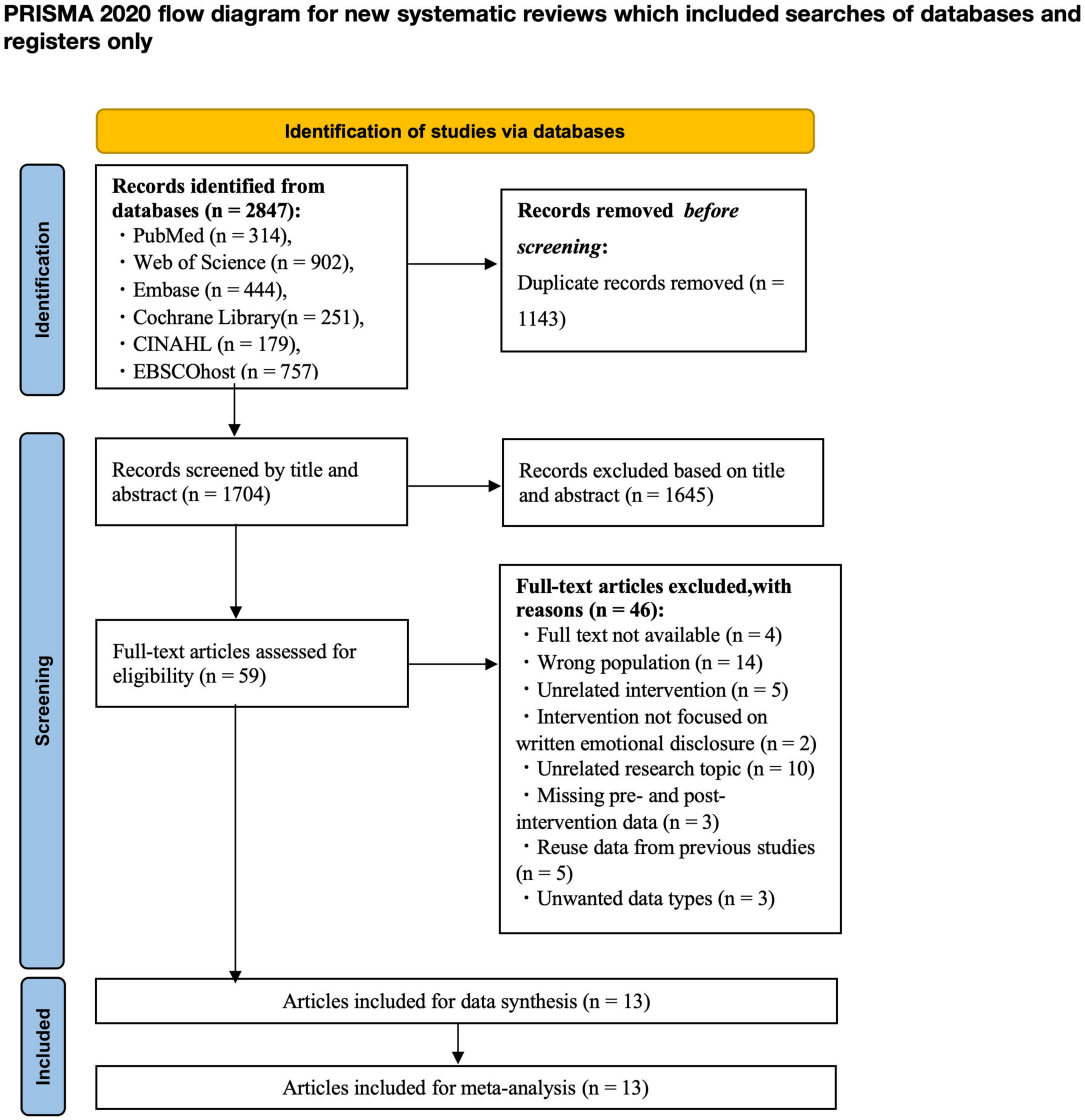
PRISMA flowchart.

Titles and abstracts were reviewed independently by two authors (YW and TX). In cases of disagreement, a third author (YT) was consulted. The PRISMA flowchart (Figure 1) summarizes the screening process for identifying, including, and excluding studies at each stage and provides the number of screenings and reasons for exclusion.

### 2.4 Data collection process

Author (YW) extracted the data and the other author (TX) verified the accuracy and completeness of the extracted data. In the face of uncertainty, the two authors discussed with each other and finally reached an agreement. Among the information obtained were: Study characteristics (authors, year of publication, country, and study design). Participant characteristics included sample size, age, gender, eating disorder diagnoses, or risk factors for eating disorders. Intervention details (form of writing intervention, number and duration of writing sessions, control group details, outcome measures and assessment time points). Supplementary Table 2 contains details about the writing contents of the experimental and control groups, time points for assessment, and follow-up rates.

### 2.5 Types of outcome measures

#### 2.5.1 Primary outcome

The primary outcome measure was eating disorder symptoms. The severity of eating disorder symptoms was gauged through a standardized self-report questionnaire or clinical rating scale. These scales primarily encompassed the Eating Disorders Examination Questionnaire (EDE-Q) (45), Eating Disorders Examination (EDE) (46) interview, Eating Disorders Scale (EDI) (47), and Eating Disorders Diagnostic Scale (EDDS) (48).

#### 2.5.2 Secondary outcomes

The secondary outcomes of this paper pertain to mental health, body image issues and the negative affect. Mental health levels were determined using standard questionnaires that evaluated depression, anxiety, and others. Typical measures comprise the Hospital Anxiety and Depression Scale (HADS) (49). Body image was assessed by self-report questionnaires such as the Body Shape Questionnaire (BSQ) (50), the Satisfaction and Dissatisfaction with Body Parts Scale (SBPDS) (51), and the Ideal Body Stereotype Scale (IBSS) (52). Negative affect was measured by the Positive Affect and Negative Affect Scale (PANAS) (53).

### 2.6 Quality assessment

Two authors (YW and XC) independently used the Cochrane Risk of Bias version 2 (RoB 2) tool to evaluate the quality of bias in randomized trials by evaluating the quality of the 13 articles screened. The risk of bias was assessed through five domains: bias in the randomization process, deviations from the intended interventions, missing outcome data, and bias in the measurement of the outcome, with the corresponding entries rated as “Yes, Y,” “Probably Yes, PY,” “Probably No, PN,” “No, N,” or “No Information, NI.” Overall bias would be judged as “Low,” “High” or “Some concerns.”

### 2.7 Data analysis

This paper will provide a narrative summary of the features, interventions, and outcomes of this study. Depending on the heterogeneity of the studies, either fixed or random effects models will be utilized for meta-analysis. Estimate the effect measures of the primary outcome. Studies presenting a high risk of bias will be excluded from the sensitivity analysis. *I^2^* will be employed to quantify heterogeneity. In case of significant heterogeneity, subgroup analyses will be conducted to identify potential sources (such as the age of the study population or the follow-up time, etc.). If an adequate amount of research is available, separate meta-analyses will be carried out for subgroups based on the category of eating disorders, the age of the participants, gender, and the form of intervention. Additionally, studies with missing outcome data are evaluated to determine whether appropriate approaches, such as intention-to-treat analyses using inferential methods, are employed to address the missing data. Studies that fail to account for missing data adequately will be regarded as having a greater risk of bias and will be excluded from meta-analysis.

#### 2.7.1 Heterogeneity

Review Manager Software version 5.4 was used for statistical analysis of data. To estimate intervention effects for continuous variables measured by various scales, standardized mean differences (SMDs) along with 95% confidence intervals (CIs) were utilized. The heterogeneity among all included studies was evaluated using the I-squared statistic (*I^2^*). It assesses the extent of heterogeneity, with a value of 0% indicating no observed heterogeneity, 25% representing low heterogeneity, 50% signifying moderate heterogeneity, and 75% indicating high heterogeneity (54). When the *p* > 0.1 and the *I^2^* statistic was 50% or lower, homogeneity among studies was assumed, and a fixed-effects model was applied. Conversely, when the *p* < 0.1 and the *I^2^* statistic exceeded 50%, significant heterogeneity among outcomes was identified, prompting an analysis of the sources of heterogeneity and the adoption of a random-effects model. If the origin of the heterogeneity could not be ascertained, a descriptive analysis of the relevant outcome indicators was conducted. To assess the reliability and robustness of the pooled results, we excluded one study at a time for sensitivity analyses. When heterogeneity was substantial, subgroup analyses were conducted. If the source of heterogeneity remained unidentified, a descriptive analysis was performed to provide a qualitative overview of the outcome indicators of interest. Egger regression tests indicated statistically significant publication bias when *p* < 0.05. The meta-analysis findings were illustrated using forest plots.

#### 2.7.2 Subgroup analysis

The main outcome indicators we addressed were eating disorder symptoms, body dissatisfaction, thin ideal internalization, anxiety, depression, and negative affect. All outcome indicators could be extracted from the studies and included in the software analysis. In the meta-analysis of the outcome indicator body image dissatisfaction, we conducted subgroup analyses for the follow-up time node (1 month) due to high heterogeneity; for the thin ideal internalization indicator, subgroup analyses based on its year of publication, sample size, and occupational characteristics of the population failed to definitively find the source of heterogeneity.

#### 2.7.3 Sensitivity analysis

This study conducted sensitivity analyses on certain outcome measures to evaluate the influence of the included studies on pooled outcomes characterized by significant heterogeneity and to determine whether the synthesized results were robust. When the heterogeneity of the outcomes was high, we performed sensitivity analyses using the exclusion-by-exclusion method. Each study was excluded in turn, and then the remaining study was combined in a meta-analysis, and the changes in the combined results were observed to assess whether the results of the original meta-analysis were significantly changed by certain studies.

#### 2.7.4 Publication bias

This study measured publication bias using funnel plots and Egger regression tests for meta-analysis involving at least 10 studies (54). The presence of publication bias was assessed using Egger’s regression intercepts (55). This method involves examining the correlation between the effect size and the standard error of the effect size to determine if there is a significant correlation between the study effect size and the study precision. If there is no publication bias, the regression intercept is zero and significant results indicate the study presents publication bias. Because this study did not include more than 10 studies in any of the several outcome indicators for which analyses were conducted, a method for detecting publication bias would show limited validity; therefore, publication bias was not assessed in this study.

### 2.8 Certainty assessment

We used the GRADEprofiler 3.6 software (56) to assess the certainty of the body of evidence for outcomes. The tool considers the initial quality of evidence from randomized controlled trials and downgrades or upgrades the quality of evidence based on the rigor of the study design, consistency of the results, precision of the effect sizes, and the potential for publication bias, etc. The quality of evidence output from GRADEprofiler 3.6 is classified into four tiers, namely, high, moderate, low, and very low, and the strength of recommendations is classified into “strong recommendation” and “weak recommendation” to guide clinical decision makers to formulate more precise treatment recommendations based on the understanding of the certainty of the evidence.

## 3 Results

### 3.1 Study selection

The process of study selection involved mainly two authors (YW and TX) independently, and during the process of the evaluation, the two authors discussed and strategized to resolve any uncertainties regarding the eligibility of the studies. Any study on which they disagreed was referred to a third author (YT) to review for inclusion. The first search of the literature base generated 2,847 studies. The remaining 1704 studies were screened for title and abstract after removing 1143 duplicate studies. After title and abstract screening, 1,645 studies were excluded. This meant that 59 studies needed to be assessed by reading the full-text. Following reading the full text, 46 studies were excluded due to non-compliance. 13 studies were ultimately included in the meta-analysis. Figure 1 summarizes our process of study selection.

### 3.2 Characteristics of the study

Among the 13 studies (36–38, 41, 57–65) included in the meta-analysis (Table 1), the majority were from Western European countries (*n* = 7; 53.8%), with four from the United Kingdom (38, 41, 58, 63), two from Sweden (37, 59), and one from the Netherlands (64); some were from the Americas (*n* = 4; 30.8%), with three from the United States (36, 57, 62) and one from Canada (61); and two were from Asia (China) (65) and Oceania (New Zealand) (60), respectively. The gender of the majority of the participant population included in the studies was female (*n* = 1,074; 74.4%), with the majority of the studies targeting women with body image-related concerns (*n* = 8; 61.5%), and the inclusion criteria for only one study (64) was a confirmed diagnosis of eating disorders. One study (64) did not mention the occupations of the participating population, while the rest of the studies focused on university students, and in the studies that did mention the participating population, the majority of the participants belonged to the white ethnic group and were from the European region.

**Table 1:** The characteristics of studies.

Author, year and country	Sample	Age (Mean)	Gender	Occupation	Ethnicity	Diagnosis/risks of eating disorders	Form of EW	Number and duration of writing sessions	Comparison group specifics
Jennifer Brenton-Peters, et al. (2023) New Zealand	53	25.77 (7.99)	Male: 18 Female: 33 Transgender: 2	All students	NZ European 32.08%, Chinese 18.87%, other 24.53%, the rest are those who did not report.	Stress	Under the guidance of researchers /write by hands	10 min	Neutral writing
Danielle Arigo and Joshua M. Smyth (2012) USA	111	18.89 (1.02)	Male: 0 Female: 111	All students	Caucasian 70%, African American 7%, Hispanic/Latina 6%	Appearance concerns	Under the guidance of researchers/write by hands	Three sessions, each lasts 15 min	Neutral writing
Kheana Barbeau, et al. (2022) Canada	114	25.8(6.9)	Male: 0 Female: 126	All students	White 85.67%	Body image concerns	Under the guidance of researchers/write by e-mails	NR	Neutral writing
Anna C. Ciao, et al. (2021) USA	76	19.90 (3.17)	Male: 13 Female: 60 Transgender: 1 gender neutral: 3	All students	White 78%, Asian 6%, Multiracial 15%, Another Identity 1%	Body image concerns	Under the guidance of peer leaders/Video Intervention	Two 2-h sessions held 1 week apart, each session includes 10 min writing time	No intervention
Philippa East, et al. (2010) UK	48	33.93 (6.32)	Male: 10 Female: 38	All students	NR	Cognitive flexibility deficits	Under the guidance of researchers/write by hands	3 days, Write for 20 min each day	Neutral writing
Ata Ghaderi, et al. (2020) Sweden	148	17.4 (1.5)	NR	Students 93.2%, other 6.8%	NR	Body image concerns	Under the guidance of researchers/write by hands	The writing lasts for a month, with each writing session lasting 40 min	No intervention
Ata Ghaderi, et al. (2022) Sweden	72	33.83 (8.40)	NR	All students	NR	Body dissatisfaction	Under the guidance of researchers/write by hands	30 min	No intervention
Olwyn Johnston, et al. (2009) UK	94	28.9 (9.8)	Male: 9 Female: 71	Students 61.3%, other 38.7%	White 76.3%, other 23.7%	Negative emotions and bulimia symptoms	Under the guidance of researchers/write by e-mails	3 days, write for 20 min each day	Neutral writing
N. Kupeli, et al. (2018) UK	71	20.54 (5.18)	Male: 0 Female: 71	All students	British 32.4%, Other European 2.8%, Indian 1.4%, Bangladeshi 1.4%, Caribbean 1.4%, African 1.4%, Mixed ethnicity 1.4%, Chinese 2.8%, other 4.2%	Stress	Under the guidance of researchers/write by hands	3 days, Write for 15 min each day	Neutral writing
Eric Stice, et al. (2006) USA	249	17.0 (1.4)	Male: 0 Female: 249	All students	Asian/Pacific Islander 10%, African American 6%, Hispanic 19%, Caucasian 58%, and 7% who specified other or mixed racial heritage.	Body dissatisfaction	Under the guidance of researchers/write by hands	Three individual weekly 45-min sessions	No intervention
Dennis Relojo (2016) UK	141	19.23 (1.21)	Male: 0 Female: 140	All students	NR	Thin-ideal images	Under the guidance of researchers/write by hands	15 min	Neutral writing
Wang and Ding (2023) China	175	20.90 (1.65)	Male: 0 Female: 175	All students	Chinese 100%	Appearance-related cyberbullying	Under the guidance of researchers/write by e-mails	10 min	No intervention
Jorg Tanis et al. (2023) Netherlands	92	35.0 (11.90)	NR	NR	NR	BED, BN UFED with BED-dynamics	Under the guidance of researchers/write by e-mails	Weekly three 45-min sessions	Neutral writing

EW, expressive writing; BED, binge eating disorder; BN, bulimia nervosa; UFED, unspecified feeding or eating disorder; NR, not reported.

### 3.3 Characteristics of intervention

#### 3.3.1 Form, session frequency, and duration of WED

Of the 13 studies included, almost all of the forms of written emotional disclosure were conducted in a researcher-led process, where participants were guided by the researcher according to the writing manual developed by Pennebaker (66). Only one study mentioned a peer-led form of writing (36). The majority of the studies (37, 38, 41, 57, 59, 60, 62, 63) encouraged participants to engage in handwriting at the time of the intervention (*n* = 8; 61.5%). The remaining five studies delivering the intervention via a media format (36, 58, 61, 64, 65). The duration of all writing interventions was mainly concentrated between 10 and 45 min, and the majority of the studies had three writing sessions during the study period, with some of them not mentioning the specific number and duration of the interventions.

#### 3.3.2 Content of WED

In all of the studies, the written emotional disclosure intervention was specified as “encouraging participants to write about their emotional state while experiencing symptoms related to the eating disorder.” The content of the writing interventions in 13 studies was categorized into the following three types: (1) Participants wrote in detail about their own emotional experiences when dealing with conditions related to their physical problems; (2) Participants wrote a letter of gratitude or encouragement from another person’s point of view (e.g., imagining themselves writing a letter of gratitude or encouragement from a friend’s point of view or from the perspective of the macrocosm, or by watching a movie and engaging in reflective writing, wrote about the emotional experiences after the movie, etc.); (3) Encouraging participants to describe moments of strong, positive emotional experiences for themselves and to write what it would be like to fantasize about themselves in that moment. Among the 13 studies, the control group essentially took a neutral writing approach, with writing that consisted of merely describing a superficial topic or describing a milestone dietary goal and did not include describing one’s emotional experience. Five of the studies had blank control groups (i.e., there was no intervention for control participants) (36, 37, 57, 59, 65). However, one of the studies added to the blank control group that those who met the criteria for anorexia nervosa, bulimia nervosa, or binge eating disorder at any follow-up assessment in the control group were referred to treatment (57).

### 3.4 Risk of bias in studies

**Figure 2:**
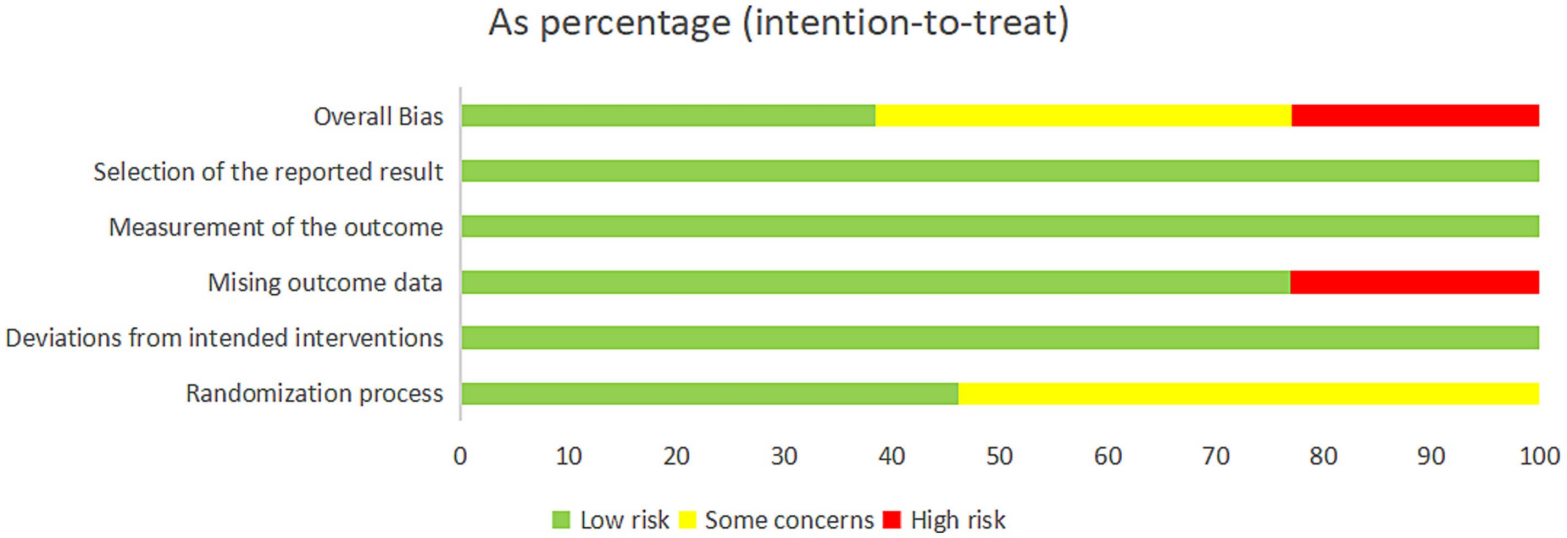
Risk of bias summary chart.

**Figure 3:**
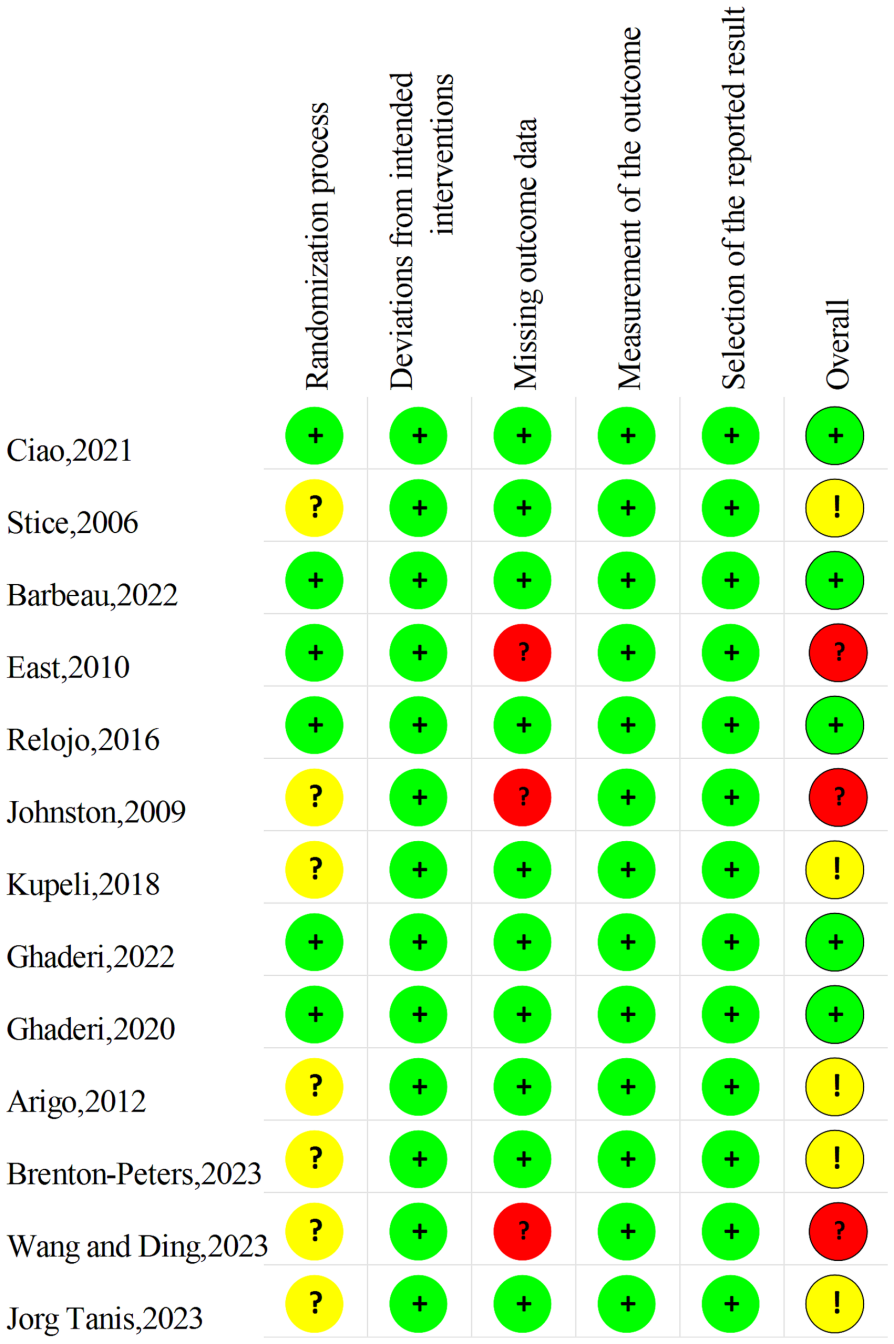
Risk of bias table.

Two authors (YW and XC) performed a risk assessment of the 13 included studies, the agreement was 80%, and the differences in the assessments were subsequently discussed and revised. The results are shown in the Figures 2, 3. Since few of the included study mentioned information related to allocation concealment, these studies were assessed as “some concerns” when performing the risk of bias assessment of the randomization process. As for the risk of bias, five studies ultimately assessed as “low” (38.5%), five as “some concerns” (38.5%), and three as “high” (23.1%).

### 3.5 Meta-analysis results

Regarding the eating disorder symptoms, eight studies employed diverse eating disorder screening questionnaires (38, 41, 57, 58, 61, 62, 64, 65); Five studies gauged body dissatisfaction by different scales (36, 37, 57, 59, 65); Four studies utilized different versions of the Ideal Body Stereotype Scale to assess the internalization of the ideal body (36, 37, 57, 63). Concerning the measurement of mental health outcomes, two studies utilized the identical anxiety and depression scales (38, 58), and four studies employed distinct negative affect scales (36, 37, 57, 65). Baseline analyses were comparable and data could be merged for meta-analysis.

#### 3.5.1 Eating disorder symptoms

**Figure 4:**
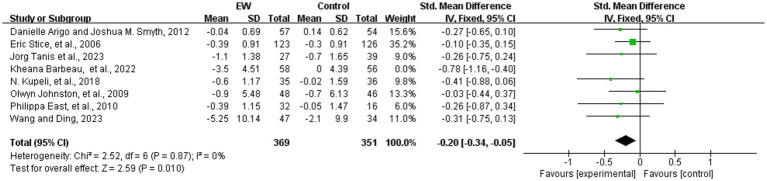
Meta-analysis about the effect of WED on alleviating eating disorder symptoms.

Eight studies analyzed the effect of WED therapy in participants with eating disorders (38, 41, 57, 58, 61, 62, 64, 65). A total of 720 study participants were included. The eight studies were scored using different scales to measure eating disorder symptoms, and SMD were utilized in the subsequent analyses. The results showed low heterogeneity (*p* = 0.17, *I^2^* = 33%), and a fixed-effect model was used to meta-analysis. The robustness of the results was tested by sensitivity analysis, and one study was excluded (61). After the group heterogeneity disappeared (*p* = 0.87, *I^2^* = 0%), meta-analysis showed that the difference in the scores of eating disorder symptoms was statistically significant (SMD = −0.20, 95% CI [−0.34, −0.05], *Z* = 2.59, *p* = 0.01) (see Figure 4).

#### 3.5.2 Body dissatisfaction

**Figure 5:**
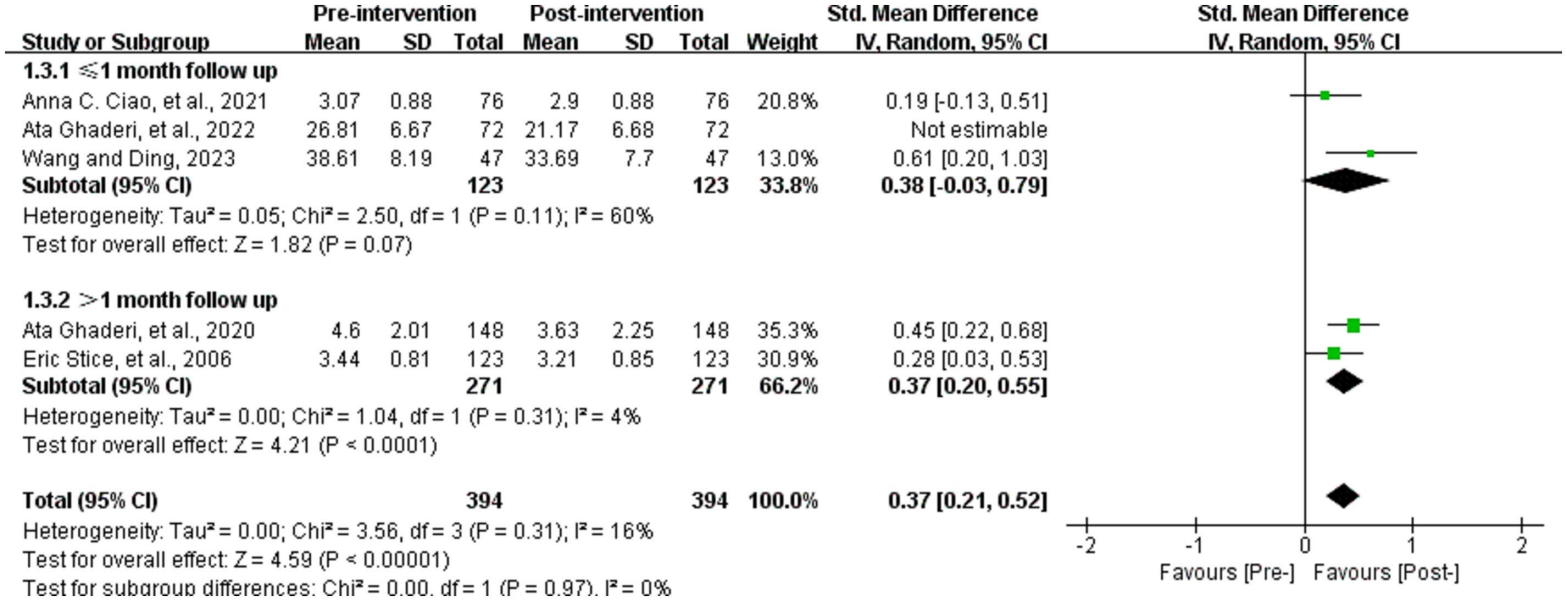
Meta-analysis about the effect of WED on alleviating body dissatisfaction.

Five studies analyzed the effect of WED therapy on body dissatisfaction in the study population (36, 37, 57, 59, 65). The five studies examined a total of 394 study participants, who were scored with diverse scales to measure the level of body dissatisfaction before and after the intervention. The outcome was analyzed by utilizing SMD, which showed a high degree of heterogeneity (*p* = 0.04, *I^2^* = 60%). Use randomized effects model for meta-analysis, subgroup analysis by length of follow-up (with 1 month as the cut-off) showed that heterogeneity still existed within the group with ≤1 month of follow-up (*p* = 0.008, *I^2^* = 74%). We performed sensitivity analysis and eventually discovered that removing the study of Ghaderi et al. (59) showed no statistically significant results although within-group heterogeneity was reduced (*p* = 0.07, *I^2^* = 60%), which may indicate that the follow-up time node is one of the factors contributing to the occurrence of heterogeneity. There may be multiple sources of heterogeneity in this result, and further search for sources of heterogeneity is needed. Statistically significant differences were found between scores of body dissatisfaction before and after the intervention (SMD = 0.37, 95% CI [0.21, 0.52], *Z* = 4.59, *p* < 0.001) (Figure 5).

#### 3.5.3 Thin ideal internalization

**Figure 6:**
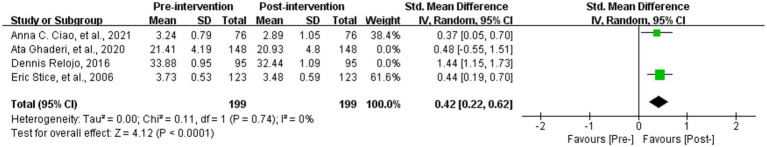
Meta-analysis about the effect of WED to mitigate the thin ideal internalization.

Four studies analyzed the effect of WED on the thin ideal internalization in the study population (36, 37, 57, 63). A total of 442 study participants were available, and the four studies were analyzed by utilizing SMD using different scales to gauge the thin ideal internalization. The results showed a high degree of heterogeneity (*p* < 0.001, *I^2^* = 93%). The meta-analysis used a random-effects model. After the sensitivity analysis, we removed two studies and the heterogeneity disappeared (37, 63) (*p* = 0.74, *I^2^* = 0%), and the results showed that the difference between the scores of the thin ideal internalization in study population before and after the intervention was statistically significant (SMD = 0.42, 95% CI [0.22, 0.62], *Z* = 4.12, *p* < 0.001), as shown in Figure 6.

#### 3.5.4 Anxiety

**Figure 7:**
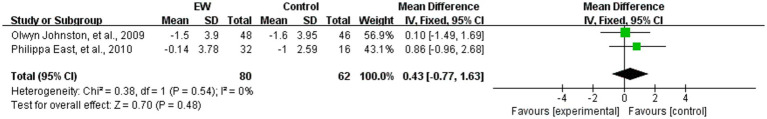
Meta-analysis of the effect of a WED intervention to alleviate anxiety.

Two studies analyzed the effect of WED on anxiety scores in the study populations (38, 58). A total of 142 study participants were included. The two studies used the Hospital Anxiety and Depression Scale (HADS) and were analyzed using MD, which showed no heterogeneity (*p* = 0.54, *I^2^* = 0%). Meta-analysis was applied using a fixed-effects model, which showed the following results: The difference between anxiety scores was not statistically significant (MD = 0.43, 95% CI [−0.77, 1.63], *Z* = 0.70, *p* = 0.48) (see Figure 7).

#### 3.5.5 Depression

**Figure 8:**
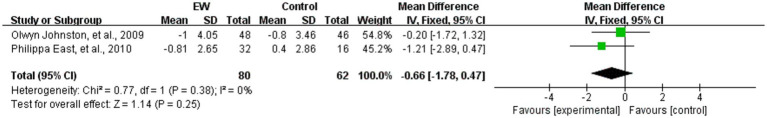
Meta-analysis about the effect of a WED intervention to alleviate depression.

Two studies analyzed the effect of WED on depression scores in the study populations (38, 58). A total of 142 study participants were included. These two studies used the Hospital Anxiety and Depression Scale (HADS), which was analyzed using MD, and the results showed that there was no heterogeneity (*p* = 0.38, *I^2^* = 0%). Meta-analysis was performed by using a fixed-effects model. The difference in depression scores was not statistically significant (MD = −0.66, 95% CI [−1.78, 0.47], *Z* = 1.14, *p* = 0.25) (see Figure 8).

#### 3.5.6 Negative affect

**Figure 9:**
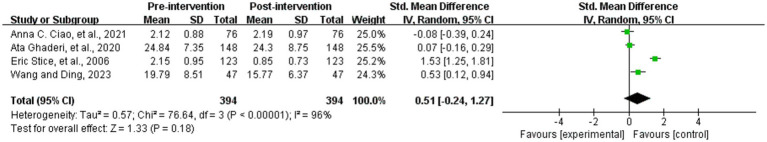
Meta-analysis about the effect of WED to reduce negative affect.

Four studies analyzed whether the WED intervention had an effect on negative affect in the study population (36, 37, 57, 65). A total of 394 study participants were included the four studies used different scales for negative affect, and were analyzed using SMD, with a large degree of heterogeneity (*p* < 0.001, *I^2^* = 96%). The random-effects model was used for meta-analysis, which showed that the difference between the negative affect scores in study population before and after the intervention was not statistically significant (SMD = 0.51, 95% CI [−0.24, 1.27], *Z* = 1.33, *p* = 0.18) (see Figure 9).

### 3.6 Publication bias

Publication bias was not assessed because there were fewer than 10 studies in each of the outcome metrics included in this study and the validity of detecting publication bias was limited, which limited our ability to assess the impact of potential publication bias. Therefore, we believe that caution should be maintained in interpreting the results of the study, and future studies need to incorporate a wider range of literature, including unpublished studies, to further enhance the robustness and credibility of the findings.

### 3.7 Certainty of evidence

**Figure 10:**
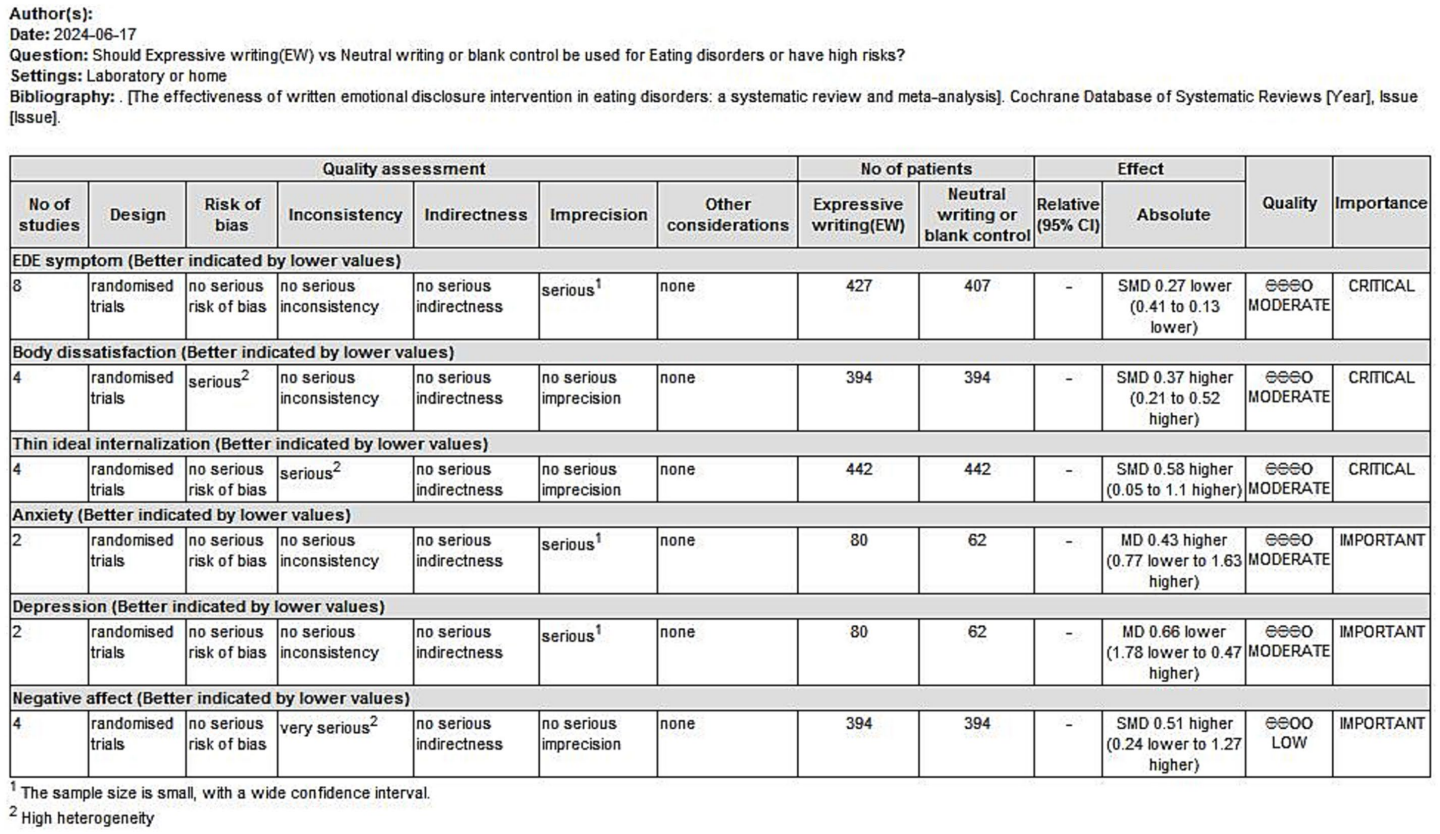
The certainty of evidence.

We evaluated the body of evidence for the outcome indicators involved in the study on the level of evidence, and the results are shown below (Figure 10). This chart shows the credibility of the evidence for eating disorder symptoms, body dissatisfaction, thin ideal internalization, anxiety, depression and negative affect, with eight randomized controlled trials (RCTs) for eating disorder symptoms, four RCTs each for body dissatisfaction and thin ideal internalization, two RCTs each for anxiety and depression, and four RCTs for negative affect. With the exception of negative affect, all of the other studies show a moderate level of evidence credibility.

## 4 Discussion

### 4.1 Potential mechanisms of WED to improve eating disorder symptoms

The results of the meta-analysis showed that WED for people with eating disorders or for those with high risk factors for developing such disorders significantly improved their eating disorder symptoms, which is consistent with the results of the Frayne et al. (40). The potential mechanisms by which written emotional disclosure interventions improve eating disorder symptoms may involve inhibitory and cognitive processes. Individuals’ failure to actively talk about important psychological phenomena can be viewed as a form of active inhibitory behavior acting on the autonomic and central nervous system activity, which is chronic low-level stress (67). Pennebaker et al. (32) showed that WED can help participants to voluntarily disclose emotional or traumatic experiences, reduces inhibitory behaviors, thereby alleviating stress. After the release of stress, individuals experience relief from negative emotions, which enables them to reassess and understand the negative experiences and emotions associated with eating disorders (58). This capacity helps to reduce the excessive focus on and sense of control over food, thereby alleviating the symptoms of eating disorders. Thus, WED interventions can be used as an effective psychotherapeutic modality in the eating disorder community and across a wide range of populations.

### 4.2 Impact of WED on body image issues

Meta-analysis results from this study showed that the WED intervention was able to relieve body dissatisfaction and thin ideal internalization among eating disorder patients and those with high-risk factors developing eating disorders, which is similar to the findings of Stice as well as Rodriguez et al. (68, 69). Melnyk et al. (70) concluded that eating disorders are associated with body image issues. And, improving body image issues can play a role in eating regulation (71). This process may involve emotion and cognition. Research has concluded that WED provides individuals with access to emotional release and helps them deal with negative emotions associated with body image (72). Reassessing and understanding one’s body image by writing about one’s feelings and experiences (73). Self-compassionate expressive writing with a body focus helps people with eating disorders to self-accept and diminish body dissatisfaction by reducing the pursuit of an ideal body image (61).

Meta-analysis showed the high level of heterogeneity, we conducted a heterogeneity analysis. We looked for multiple subgroups, but with unsatisfactory results. After sensitivity analysis, the heterogeneity disappeared after excluding the two studies that had a large impact on the results, and therefore these two studies were considered as possible major sources of heterogeneity. Further analysis revealed that the two excluded studies were of low quality and lacked some specific information (e.g., gender and ethnicity) to be used in the analysis of heterogeneity and could be the source of heterogeneity.

### 4.3 Role of WED on anxiety, depression, and the negative affect

The results of the meta-analysis in this study revealed that the WED intervention had no effect on the levels of anxiety and depression, as well as on the negative affect of the study population. However, research has shown that WED intervention was effective in reducing levels of anxiety and depression in the eating disorder population long after the intervention (38). Another result showed that WED applied to cancer caregivers may improve their adverse conditions of burden, anxiety, and depression (74). It is evident that WED is valid in improving conditions such as anxiety and depression, but the effectiveness may be different due to the different study populations. This may also be attributed to the condition of eating disorder disease, which complicates the negative psychological emotions in this population. Therefore, the role of WED interventions on anxiety, depression, and negative affect in eating-disordered populations cannot be concluded with certainty, more careful consideration is needed.

### 4.4 Comparative effectiveness of emotional expression interventions

This meta-analysis has focused on examining the effectiveness of WED interventions for eating disorders (EDs), with results indicating that WED has certain effects in alleviating ED symptoms. However, the realm of emotional expression extends beyond writing, and other forms of emotional expression interventions, such as verbal therapy and art therapy, are also worthy of attention. Verbal disclosure, by providing immediate feedback and interpersonal interaction, may be more conducive for some individuals to deeply understand and process their emotions. Empirical studies have provided evidence to corroborate that verbal disclosure can facilitate emotional expression and cognitive restructuring, which are crucial for the recovery process in eating disorders. Several studies (75, 76) have investigated the role of recovered eating disorder patients as therapists, using verbal self-disclosure in treatment. Results showed that sharing their insights helps patients better understand recovery, reduces shame, and alleviates eating disorder symptoms. This approach effectively encourages patients to self-disclosure, yielding positive therapeutic effects.

Art therapy can enhance an individual’s emotional regulation capabilities and reduce stress and anxiety, both of which are closely related to emotional states associated with ED symptoms. A systematic review conducted by Griffin et al. (77) demonstrated that art therapy enhances self-esteem and alleviates anxiety in individuals with eating disorders by fostering self-expression, increasing self-awareness, and cultivating new perspectives and a sense of pride. Also, the study by Trably et al. (78) showed that art therapy has the potential to enhance specific dimensions of individuals with eating disorders: in particular, music therapy has been shown to significantly alleviate post-meal anxiety, while dance movement therapy can markedly reduce body dissatisfaction.

Nevertheless this meta-analysis did not encompass direct comparisons between these interventions, mainly due to the scarcity of eligible studies and the heterogeneity in methodologies and outcome measurements. Consequently, we advocate for future research to investigate the comparative efficacy of WED against other emotional expression therapies. This exploration is crucial for discerning their respective benefits and suitability in ED treatment. Such analyses would deepen our comprehension of intervention efficacy and uncover individual propensities and reactions to various therapeutic approaches, laying the groundwork for tailored treatment strategies.

### 4.5 Future perspectives on WED

The majority of the current research has focused on examining the effects of WED on females and adolescents in Western Europe, so future research could focus more on populations of different ages, genders, geographic areas, and cultural backgrounds. In addition, changes in the environment in which WED is implemented could be considered in the future. Currently, most implementation sites are mainly in laboratories and medical facilities. According to the meta-analysis by Frattaroli, compared to a controlled laboratory setting, WED is more effective at home (79). Also, Gripsrud et al. (80) found in interviews with breast cancer patients that it was easier for them to complete their writing at home.

The frequency, duration, and setting of WED implementation, as well as the instructor who delivered the intervention, were not standardized in the current study. The duration of WED implementation was 15–30 min in most studies, but a few studies fell outside this time range. Most studies chose to conduct WED interventions in the laboratory. The pre-intervention instructors were mostly researchers, with a few adopting peer instruction. Therefore, in the future, consideration needs to be given to the development of a harmonized guideline for clinicians as well as practitioners on the application of WED in the ED population. This guide could focus on describing the frequency and duration of writing, detailing the topics and types of writing, noting the context in which the writing is implemented, considering the interaction of ED population characteristics with writing (e.g., gender, age, geographic region, etc.), and designating the instructor who will implement the intervention.

### 4.6 Limitations and prospects

Several limitations of this meta-analysis need to be mentioned. First, the quality of the included 13 studies was low. Most of the studies only mentioned randomization without elaborating on the specific methods, and very few of them had reference to allocation concealment and specific blinding information. This lack of detail may have some impact on the results of risk of bias assessment. Second, the majority of the study populations were college students and women, the baseline level of eating disorder symptoms in these participants may not be consistent, increasing the risk of selection bias. Third, this study may exist the risk of publication bias. This may be related to the fact that the included studies focused on observing groups of women in Western countries. This limitation is primarily attributed to the existing focus on female patients within the field of eating disorders and the geographical scope of the research. Further attention needs to be paid to individuals from different cultural backgrounds and of different genders in relevant studies in the future, thereby expanding the sample size and validating the consistency of WED’s therapeutic efficacy across various populations. Furthermore, some studies may have obtained more significant results as they used specific scales. Several of the outcome indicators were not scored using a uniform scale, which may have limited our ability to synthesize the results of the studies. And it may be at potential risk of publication bias.

Additionally, some of the outcome indicators were highly heterogeneous and may be associated with sample size, baseline level of the study population, age, gender, cultural background, and duration of follow-up. However, the limited information provided by the included studies resulted in this study not being able to analyze the sources of heterogeneity in depth. In future studies, we will pre-define possible subgroups at the study design stage and include these subgroups (e.g., gender, age, and region) in meta-analyses, even if some information is missing, and try to obtain data from other sources or use alternative indicators. We will also improve the data extraction process, handle missing data more transparently, and select appropriate statistical methods to better understand and account for heterogeneity.

Moreover, the absence of direct comparisons between WED and other emotional expression interventions in our systematic review and meta-analysis. This is primarily due to the diversity of included studies and limitations in data availability. Specifically, the heterogeneity in methodologies, participant characteristics, and outcome measures across different studies complicate direct comparisons. Furthermore, the limited number of studies in the existing literature that directly compare WED with other emotional expression interventions restricts our ability to conduct a comprehensive comparison. Therefore, we recommend that future research should address these limitations to more accurately assess the impact of various intervention measures on eating disorder symptoms.

## 5 Conclusion

The available evidence from this study suggests that WED is effective in alleviating eating disorder symptoms and body image problems, but there is inadequate evidence in relieving depression, anxiety, and negative affect. Hence, future researches on the impact of WED on depression, anxiety, and negative affect in eating disordered populations should be appropriately emphasized to explore its role.

## Data Availability

The original contributions presented in the study are included in the article/Supplementary material, further inquiries can be directed to the corresponding author.
