# Supplementary material for: Effectiveness of written emotional disclosure interventions for eating disorders: a systematic review and meta-analysis
Source: Front Nutr. 2024 Dec 10;11:1476956. doi: 10.3389/fnut.2024.1476956 (PMC11667891; doi:10.3389/fnut.2024.1476956)
Supplement: Supplementary file 1 [file Table_1.DOCX]

**Search strategy**.

**1. Search strategy for potential eligible studies:**

1. **Search strategy for MEDLINE via PubMed**

| **Number** | **Search terms** |
| --- | --- |
| #1 | Writing[Title/Abstract] OR "expressive writing"[Title/Abstract] OR "written disclosure"[Title/Abstract] OR "emotion expression"[Title/Abstract] OR "emotion disclosure"[Title/Abstract] OR "Written emotional disclosure"[Title/Abstract] OR diary[Title/Abstract] OR Self-Compassion*[Title/Abstract] |
| #2 | "Feeding and Eating Disorders"[Mesh] OR "Binge-Eating Disorder"[Mesh] OR eating disorder*[Title/Abstract] OR disordered eat*[Title/Abstract] OR "anorexia nervosa"[Title/Abstract] OR "bulimia nervosa"[Title/Abstract] |
| #3 | #1 AND #2 |

1. **Search strategy for EMBASE**

| **Number** | **Search terms** |
| --- | --- |
| #1 | writing:ab,ti OR 'expressive writing':ab,ti OR 'written disclosure':ab,ti OR 'emotion expression':ab,ti OR 'emotion disclosure':ab,ti OR 'written emotional disclosure':ab,ti OR diary:ab,ti OR 'self compassion*':ab,ti |
| #2 | 'eating disorder'/exp |
| #3 | 'eating disorder*':ab,ti OR 'disordered eat*':ab,ti OR 'anorexia nervosa':ab,ti OR 'bulimia nervosa':ab,ti |
| #4 | #2 OR #3 |
| #5 | #1 AND #4 |

1. **Search strategy for Web of Science**

| **Number** | **Search terms** |
| --- | --- |
| #1 | TS=(Writing OR "expressive writing" OR "written disclosure" OR "emotion expression" OR "emotion disclosure" OR "Written emotional disclosure" OR diary OR Self-Compassion*) |
| #2 | TS=("Feeding and Eating Disorders" OR "Binge-Eating Disorder" OR eating disorder* OR disordered eat* OR "anorexia nervosa" OR "bulimia nervosa") |
| #3 | #1 AND #2 |

1. **Search strategy for Global Health, Cumulative Index to Nursing and Allied Health Literature (CINAHL), EBSCO**

| **Number** | **Search terms** |
| --- | --- |
| #1 | TI ( Writing OR "expressive writing" OR "written disclosure" OR "emotion expression" OR "emotion disclosure" OR "Written emotional disclosure" OR diary OR Self-Compassion* ) OR AB ( Writing OR "expressive writing" OR "written disclosure" OR "emotion expression" OR "emotion disclosure" OR "Written emotional disclosure" OR diary OR Self-Compassion* ) |
| #2 | TI ( "Feeding and Eating Disorders" OR "Binge-Eating Disorder" OR eating disorder* OR disordered eat* OR "anorexia nervosa" OR "bulimia nervosa" ) OR AB ( "Feeding and Eating Disorders" OR "Binge-Eating Disorder" OR eating disorder* OR disordered eat* OR "anorexia nervosa" OR "bulimia nervosa" ) OR SU (Eating Disorder) |
| #3 | #1 AND #2 |

1. **Search strategy for Cochrane library**

| **Number** | **Search terms** |
| --- | --- |
| #1 | MeSH descriptor: [Feeding and Eating Disorders] explode all trees |
| #2 | MeSH descriptor: [Binge-Eating Disorder] explode all trees |
| #3 | ("Feeding and Eating Disorders" OR "Binge-Eating Disorder" OR eating disorder* OR disordered eat* OR "anorexia nervosa" OR "bulimia nervosa"):ti,ab,kw |
| #4 | (Writing OR "expressive writing" OR "written disclosure" OR "emotion expression" OR "emotion disclosure" OR "Written emotional disclosure" OR diary OR Self-Compassion*):ti,ab,kw |
| #5 | #1 OR #2 OR #3 |
| #6 | #4 AND #5 |
